# Chromatography paper strip sampling of enteric adenoviruses type 40 and 41 positive stool specimens

**DOI:** 10.1186/1743-422X-2-6

**Published:** 2005-02-10

**Authors:** Kalina T Zlateva, Piet Maes, Mustafizur Rahman, Marc Van Ranst

**Affiliations:** 1 Laboratory of Clinical & Epidemiological Virology, Department of Microbiology and Immunology, Rega Institute for Medical Research, University of Leuven, Leuven, Belgium; 2 Laboratory of Virology, ICDDR, B: Center for Health and Population Research, Dhaka, Bangladesh

**Keywords:** enteric adenoviruses

## Abstract

**Background:**

The enteric subgroup F adenoviruses type 40 (Ad40) and 41 (Ad41) are the second most important cause of acute infantile gastroenteritis after rotaviruses. Repeated community outbreaks have been associated with antigenic changes among the Ad40 and Ad41 strains due to host immune pressure. Therefore large field epidemiological surveys and studies on the genetic variations in different isolates of Ad40 and Ad41 are important for disease control programs, the design of efficient diagnostic kits and vaccines against subgroup F adenoviruses. A novel method using sodium dodecyl sulphate SDS/EDTA-pretreated chromatography paper strips was evaluated for the collection, storage and shipping of Ad40/41 contaminated stool samples.

**Results:**

This study shows that adenoviral DNA can be successfully detected in the filter strips by PCR after four months storage at -20°C, 4°C, room temperature (20–25°C) and 37°C. Furthermore no adenoviral infectivity was observed upon contact with the SDS/EDTA-pretreated strips.

**Conclusions:**

Collecting, storing and transporting adenovirus type 40 and 41 positive stool samples on SDS/EDTA-pretreated chromatography filter strips is a convenient, biosafe and cost effective method for studying new genome variants and monitoring spread of enteric adenovirus strains during outbreaks.

## Background

Enteric adenoviruses (EAds) are considered to be the second most important causative agent of acute infantile gastroenteritis after rotaviruses. The fastidious subgroup F adenoviruses type 40 (Ad40) and 41 (Ad41) account for the majority of cases of severe acute diarrhea in children less than 2 years of age [1,2]. These viruses usually cause sporadic infantile gastroenteritis, but they have also been implicated in outbreaks and nosocomially acquired diarrhea [3-5]. The course of the disease is mild and self-limiting in most cases, but in immunocompromised patients these infections are associated with an increased morbidity and prolonged hospitalization [6,7]. Repeated community outbreaks and shift in the prevailing subgroup F adenovirus type have been associated with antigenic changes among the Ad40 and Ad41 strains due to host immune pressure [8-12]. Therefore, large field epidemiological surveys and studies on the genetic variations in different isolates of Ad40 and Ad41 are important for disease control programs, the design of efficient diagnostic kits and vaccines against subgroup F adenoviruses. For these purposes stool samples need to be collected, stored and transported to reference laboratories for genetic analysis. In many developing countries and remote areas, collection and storage of samples for laboratory diagnosis is difficult due to a restricted infrastructure. Moreover field conditions may limit the handling, transportation and refrigeration of the specimens.

Previous studies have demonstrated the application of different filter papers for the collection and storage of blood [13], saliva [14] and stool [15] samples for further analysis. Filter paper sampling has been successfully used for screening studies of rotaviruses [16], noroviruses [17], human herpesviruses 6 and 7 [14], human immunodeficiency virus [13,18], hepatitis C virus [19], measles virus [20] and others viruses.

This study describes the use of SDS/EDTA-pretreated filter paper strips in collection, transportation and storage of adenovirus type 40/41 positive stool samples for subsequent genetic analysis.

## Results and Discussion

In the current study we describe the use of chromatography paper strips for the collection, transportation, and storage of EAds type 40/41 positive stool samples. In order to inactivate the adenoviruses and other microorganisms upon contact with the strips, the latter were pretreated with SDS, a surfactant with protein denaturising ability. This can allow safe transportation of the strips without extensive biohazard precautions. To protect the viral DNA from degradation by deoxyribonucleases (DNases) the chromatography strips were also preincubated with EDTA, and Tris-HCl. EDTA chelates magnesium ions, a necessary co-factor for most nucleases and the weak organic base Tris-HCl ensures the proper action of the chelating agent in binding the divalent cations.

**Figure 1:**
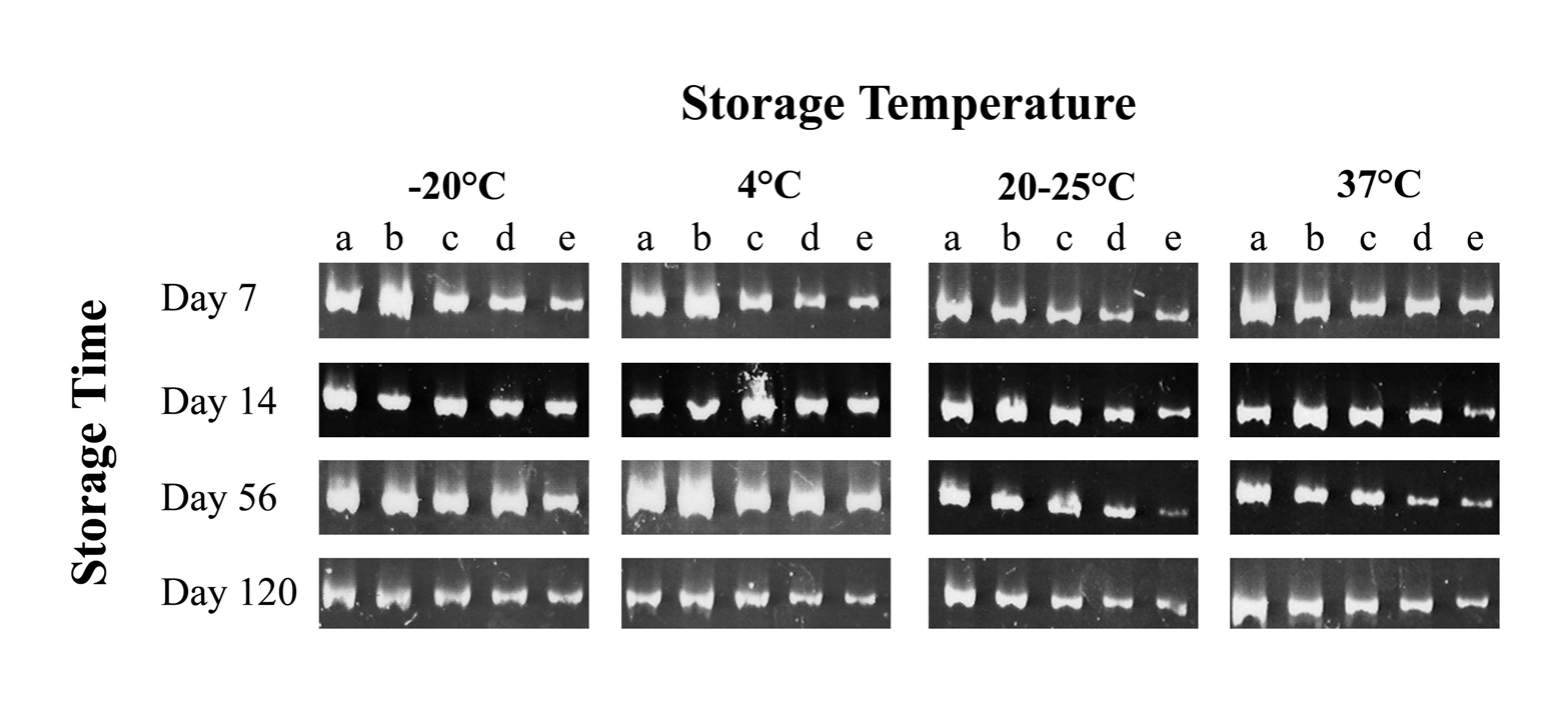
Polyacrylamide gel electrophoresis of the PCR products amplified from the DNA of the subgroup F adenovirus positive stool sample, extracted from the SDS/EDTA pretreated chromatography paper strips that have been stored at four different temperature conditions. Five tenfold dilutions of the original stool sample were tested (*a *= 1:8, *b *= 1:80, *c *= 1:800, *d *= 1:8000, *e *= 1:80,000).

A diarrheal stool sample containing 2.6 × 10^6 ^adenoviral particles per ml was serially diluted 1:8 (dilution *a*), 1:80 (dilution *b*), 1:800 (dilution *c*), 1:8000 (dilution *d*) and 1:80,000 (dilution *e*). The SDS/EDTA-pretreated filter paper strips were infected with each stool dilution and stored at -20°C, 4°C, room temperature (20 to 25°C), and 37°C. The presence of adenoviral DNA on the chromatography filter strips was detected by PCR amplification of a 301 bp fragment of the adenoviral hexone gene after storage for 7 days, 14 days, 56 days and 120 days (Figure 1).

Our results show that adenoviral DNA can remain stable even at higher than room temperature conditions for at least 4 months, indicating that the collection and storage of the infected filter strips is possible where freezers are not available.

**Figure 2:**
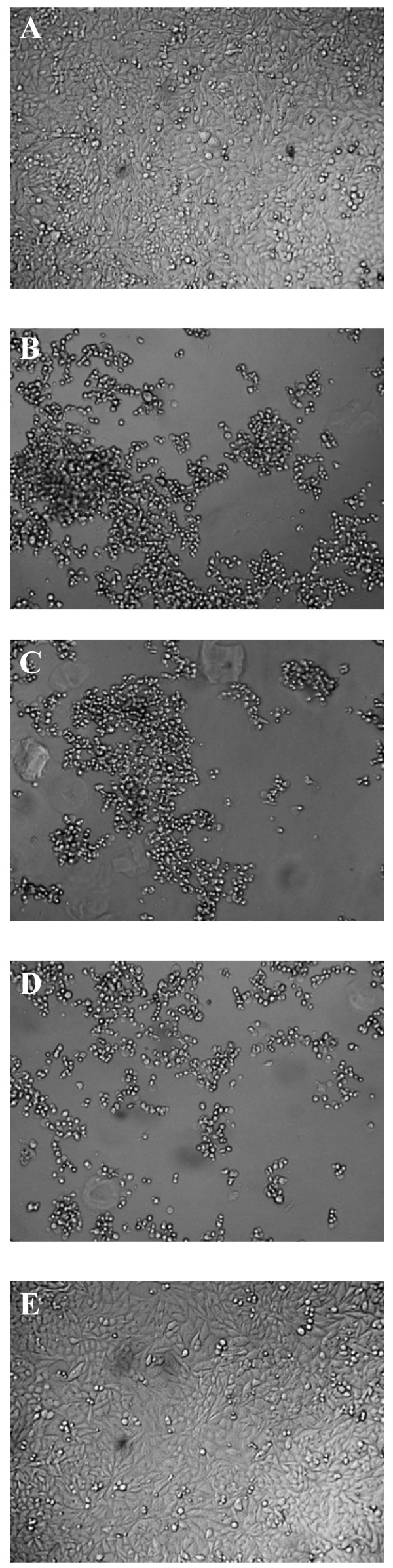
(A) Normal HeLa confluent monolayer. (B) CPE in the HeLa cells at 3 days after infection with adenovirus type 1. (C) CPE in the HeLa cells at 3 days after infection with 500 μl eluate of the infected adenovirus type 1 untreated filter paper strips. (D) CPE in the HeLa cells after 3 days infected with the dialysed adenovirus type 1 positive cell cultured sample. (E) No CPE was observed when the HeLa cells were infected with 500 μl of the dialysed eluate from the adenovirus type 1 infected SDS/EDTA-pretreated paper strip.

To be sure that the EAd40/41 contaminated filter strips are not infectious, we carried out a biosafety test to find out if any adenovirus could survive onto the SDS/EDTA-pretreated strips. Previously we showed that pathogenic bacteria such as *Vibrio cholerae*, enterotoxigenic *E. coli*, enteropathogenic *E. coli*, *Salmonelle typhimurium *and *Shigella dysenteriae*, were not able to survive on the SDS/EDTA strips [16]. The biosafety test performed in this study demonstrated that adenoviruses also lost infectivity upon contact with the SDS/EDTA strips (Figure 2). In HeLa cell line, no cytopathic effect was observed after incubation with the dialyzed eluate of the SDS/EDTA strips loaded with adenovirus type 1 (10^6 ^TCID 50/ml) after three passages of the infected cell line. The eluate of the untreated chromatography strips loaded with adenovirus type 1 caused cytopathic effect in the HeLa cell line. It can be concluded that the SDS/EDTA-pretreated strips can be used for the collection and shipping of adenovirus positive stool samples from remote areas to reference laboratories in a biosafe way.

## Conclusions

We conclude that the use SDS/EDTA-pretreated filter strips for retrieval and subsequent analysis of adenoviral DNA from EAds type 40/41 positive stool specimens is a feasible method for sample collection. The described filter paper strips facilitate collection, transport and storage of adenoviral positive stools because they are biosafe, cost effective and require minimal storage space. This study shows that adenoviral DNA can remain stable for at least 4 months at 37°C temperature conditions making this method especially attractive for field research or population screening in tropical countries where freezers are not available.

## Materials and Methods

### Chromatography paper strips

Highly absorbent (870 g of water/m^2^) Whatman grade 17 chr pure cellulose chromatography paper with thickness of 0.92 mm and a flow rate of 190 mm/30 min (Whatman, Kent, United Kingdom) was used. Strips of 80 mm × 4 mm were cut from the chromatography paper and soaked for two minutes in a solution of 2% (w/v) sodium dodecyl sulphate (SDS), 10 mM EDTA and 60 mM TrisHCl. The chromatography paper strips were left to dry overnight at room temperature. Disposable gloves were used during the preparation of the filter paper strips.

### Adenovirus sample loading on the chromatography paper strips

A diarrhea stool sample that was positive for adenovirus type 40/41 hexon antigen by the Premier Adenoclone^®^-Type 40/41 solid-phase sandwich enzyme immunoassay (Meridian Bioscience, Cincinnati, Ohio) was used for this study. The undiluted feces sample contained approximately 2.6 × 10^6^ particles per ml of stool, as calculated from a standard curve supplied with the antigen enzyme immunoassay kit. The stool sample was diluted in 1 ml (dilution 1:8) DNase/RNase free water (Sigma) and the following dilutions were used: 1:8 (dilution *a*), 1:80 (dilution *b*), 1:800 (dilution *c*), and 1:8000 (dilution *d*), and 1:80000 (dilution *e*). The pretreated chromatography strips were infected with 100 μl of the different dilutions of stool sample and were left to air dry overnight at room temperature. After complete drying, the infected strips were stored under four different temperature conditions: -20°C, 4°C, room temperature (20 to 25°C) and 37°C.

### PCR detection

Half of the strip (160 mm^2^) was used for the DNA extraction performed at the following storage time intervals: 7, 14, 56 and 120 days. The filter paper was inserted into an Eppendorf tube with 500 μl of Dnase/Rnase free water (Sigma) and thoroughly squeezed out. An aliquot of 200 μl of the squeezed eluate was used for DNA extraction using the QIAamp DNA Blood Mini Kit (Qiagen/Westburg, Leusden, The Netherlands) according to the manufacturer's instructions. A set of degenerate consensus primers (forward primer 5'-GCCSCARTGGKCWTACATGCACATC-3' and (reverse primer 5'-CAGCACSCCICGRATGTCAAA-3') were used to amplify a 301 bp fragment of the adenoviral hexone gene [21]. The PCR assay was performed with 10 μl of the extracted DNA in a 50 μl total volume, containing 0.5 μM of forward and reverse primer, 200 μM nucleotides, 2.5 mM MgCl_2_, and 1 unit Taq polymerase (Applied Biosystems, Foster City, CA). The PCR was conducted in a Geneamp PCR System 9600 thermal cycler (Applied Biosystems). The thermocycling conditions consisted of denaturation at 94°C for 3 min, followed by 35 cycles of 30 s at 94°C, 30 s at 55°C and 1 min at 72°C and 5 min of final elongation at 72°C. PCR products were visualized using polyacrylamide gel electrophoresis and ethidium bromide staining.

### Biosafety test for adenovirus

A biosafety experiment was performed to check if adenoviral particles are still infectious after contact with the SDS/EDTS-pretreated chromatography paper strips. Since Ad40 and Ad41 grow poorly in cell culture it is difficult to detect these viruses *in vivo*. Therefore adenovirus type 1 was used for the biosafety experiments. The SDS/EDTA-pretreated filter stips were first infected with 100 μl of the HeLa cell cultured adenovirus type 1 (10^6 ^TCID 50/ml) and allowed to dry at room temperature for 60 min. The strips were then placed into an eppendorf tube containing 500 μl Dulbecco's Modified Eadle Medium (DMEM) (Invitrogen, Merelbeke, Belgium) supplemented with 200 mM L-glutamine (Sigma-Aldricht, Bornem, Belgium). The strips were thoroughly squeezed in the medium and the eluate was dialyzed using 3,500-Da Slide-A-Lyzer dialysis cassettes (Pierce Biotechnology, Rockford, IL, USA) to remove the cytotoxic SDS. The dialyzed eluate was inoculated on a confluent monolayer of HeLa cells and was incubated at 37°C in a humified incubator with a 5% CO_2 _environment. Untreated strips infected with adenovirus type 1 and noninfected SDS/EDTA-pretreated strips were used as positive and negative controls respectively. The presence of cytopathic effect indicated the presence of live replicating virus on the strip. Cytopathic effects were monitored up to the third passage of the tissue culture supernatant.

## List of abbreviations

Eads – enteric adenoviruses

EDTA – ethylenediamine tetra-acetic acid

SDS – sodium dodecyl sulphate

## Competing interests

The author(s) declare that they have no competing interests.

## Authors' contributions

KZ conducted the study, carried out the experiments and wrote the manuscript. PM carried out the biosafety experiments. MR developed the filter paper strip sampling method. MVR supervised the study and revised the manuscript. All authors read and approved the manuscript.
